# External fixation via the anterior inferior iliac spine for proximal femoral fractures in young patients

**DOI:** 10.1515/med-2021-0295

**Published:** 2021-08-04

**Authors:** Qing Yang, Nong Chen, Wenqin Fu

**Affiliations:** Department of Orthopedics, QingPu Branch of Zhongshan Hospital Affiliated to Fudan University, QingPu District Central Hospital Shanghai, Shanghai, 201700, People’s Republic of China

**Keywords:** femoral fractures, external fixators, fracture fixation, internal

## Abstract

**Background and aim:**

Acute treatment of young patients with proximal femoral fracture (PFF) remains a challenge for trauma surgeons due to major fracture displacement and heavy pain in clinical practice. Traditional methods have a variety of intrinsic defaults and cannot successfully manage the requirements of young patients. Benefiting from our anatomic research, we explored a new method of external fixation for this specific trauma and evaluated its feasibility and clinical outcomes.

**Material and methods:**

Twenty-three young multiple-trauma patients with PFF were included in this study. Surgical treatment was applied using an external fixator via the anterior inferior iliac spine (AIIS). Electronic patient records, surgical characteristics, clinical outcomes, and complications were reviewed for each patient.

**Results:**

The mean surgical time was 30.3 ± 7.3 min. The mean blood loss was 25.3 ± 10.8 mL. No iatrogenic nerve palsy, pin tract infection, failure of external fixation, or bedsores were observed. The postoperative visual analog scale score was significantly lower than the preoperative score (*P* < 0.01). The mean fracture reduction rate of the femur was 58.1 ± 17.0%, and the mean degree of reduction was 13.5 ± 6.9°. The mean external fixation time was 7.6 ± 4.0 days and intramedullary nailing was performed. The mean hospital, follow-up, and healing times were 28.7 ± 8.7 days, 23.5 ± 7.9 months, and 22.8 ± 5.7 weeks, respectively. The Harris Hip Score indicated excellent or good results in 20 patients.

**Conclusion:**

Collectively, the results of this study revealed that external fixation via the AIIS is a safe, rapid, and effective method for acute treatment of PFF in young patients.

## 1 Introduction

Proximal femoral fractures (PFFs) in young patients are the result of high energy impact and often associated with major fracture displacement and heavy pain. Acute treatment of these specific fractures remains a challenge for trauma surgeons due to multisystem injuries and poor conditions during emergencies [1,2]. Traditionally, acute treatment of PFFs involves lower-limb skin or skeletal traction [3,4,5], and then intramedullary nailing is considered to be the definitive treatment in the majority of trauma patients [6,7,8,9,10]. With the extensive use of external fixators in trauma surgery, some literature has described the application of an external fixator to the PFF as the definitive treatment, but it is associated with extensive soft tissue damage, severe open fractures [11,12], and poor general conditions [13,14,15,16,17]. Traditional lower-limb traction and the aforementioned methods of external fixation still have some faults [3,4,18], including cortical defects, pin tract infection, secondary pin-tract osteomyelitis, or septic arthritis if placed intra-articularly [19]. In addition, long-term traction and unstable external fixation may result in a high risk of bedsores, pneumonia, deep vein thrombosis, and urinary tract infection [20]. With advances in implant material, surgical techniques, and anatomic research, and popularization of the concept of enhanced recovery after surgery (EARS), young patients have high requirements for reducing postoperative complications, shortening the length of the hospital stay, improving satisfaction, and accelerating recovery [21]. This presents an urgent problem for trauma surgeons to explore an ideal method of external fixation for PFF in young patients who can achieve initial stabilization, pain relief, and conversion to definitive treatment in two stages.

**Figure 1:**
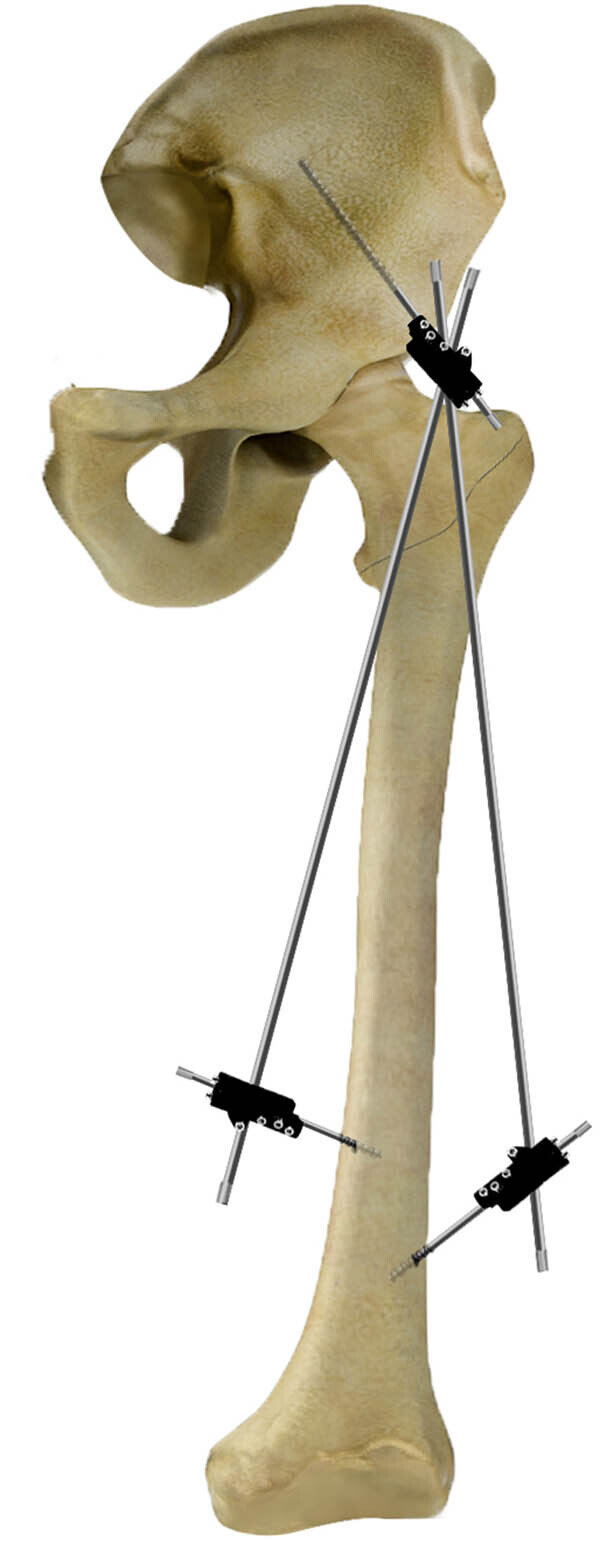
It illustrates the details of this novel external fixation method via the AIIS and distal femur.

Our previous study demonstrated that the anterior inferior iliac spine (AIIS), which is close to the proximal femur possesses, has the thickest bone mass and largest anti-pulling force. The AIIS presents an ideal site for proximal pin insertion, and the other pins are set to be inserted into the distal femur at different levels [22]. External fixation via the AIIS for PFF has predictable initial stabilization biomechanically and permits conversion to intramedullary nailing as a reliable surgical procedure clinically [2,23] when young patients’ general condition permits major surgery. Figure 1 illustrates the details of this novel external fixation method via the AIIS.

The aim of this study was to evaluate the feasibility, surgical safety, and clinical outcomes of this novel method of external fixation via the AIIS as an acute treatment option for PFF in young trauma patients.

## 2 Patients and methods

### 2.1 Study population

A prospective analysis of external fixation via the AIIS and distal femur was performed in our level I trauma center between January 2015 and December 2017. The inclusion criteria were young trauma patients (<65 years) suffering an intertrochanteric femoral fracture and/or subtrochanteric femoral fracture with major displacement. Exclusion criteria were age >65 years, isolated PFF with little displacement, hemodynamic instability, serious open fractures (Gustilo III) [24], serious medical conditions (e.g., diabetes mellitus, heart, and respiratory diseases), and serious osteoporosis.

A total of 23 young trauma patients with PFF were included and treated with external fixation via the AIIS and distal femur (Table 1). The patients consisted of 13 men (56.5%) and 10 women (43.5%). The mean patient age was 45.8 ± 14.2 years (range, 29–63 years). In 7 cases (30.4%), the right femur was involved; in 16 cases (69.6%), the left femur was involved. Only three patients had open fractures graded Gustilo I–II. The mechanisms of injury included motor vehicle accidents (*n* = 11, 47.8%) and falls (*n* = 12, 52.2%). All patients were treated with external fixation via the AIIS in emergency surgery, and the other system injuries were initially managed at the same time according to the guidelines of the Advanced Trauma Life Support (ATLS) [25].

**Table 1:** General data

Data	*n* (%)/Mean ± SD
Gender	
Male	13 (56.5)
Female	10 (43.5)
Age (years)	45.8 ± 14.2
Mechanism	
Motor vehicle accidents	11 (47.8)
Fall	12 (52.2)
Proximal femoral fractures (*)	
Left	16 (69.6)^a^
Right	7 (30.4)
Open fractures	2 (8.7)
Closed fracture	21 (91.3)

a *P* < 0.01, Left vs Right. General data of this study show nonsignificant differences except in limb sides. Values are expressed as the mean ± standard deviation or *n* (%).

* Intertrochanteric femoral fracture and/or subtrochanteric femoral fracture with major displacement.


**Ethical statement:** This study was reviewed and approved by the Institutional Review Board of the Qingpu Branch of Zhongshan Hospital affiliated with Fudan University Qingpu District Central Hospital Shanghai. This study was performed following the principles of the 1964 Declaration of Helsinki. All participants were asked to sign written consent for publication of their X-rays and photographs.

### 2.2 Surgical procedures

**Figure 2:**
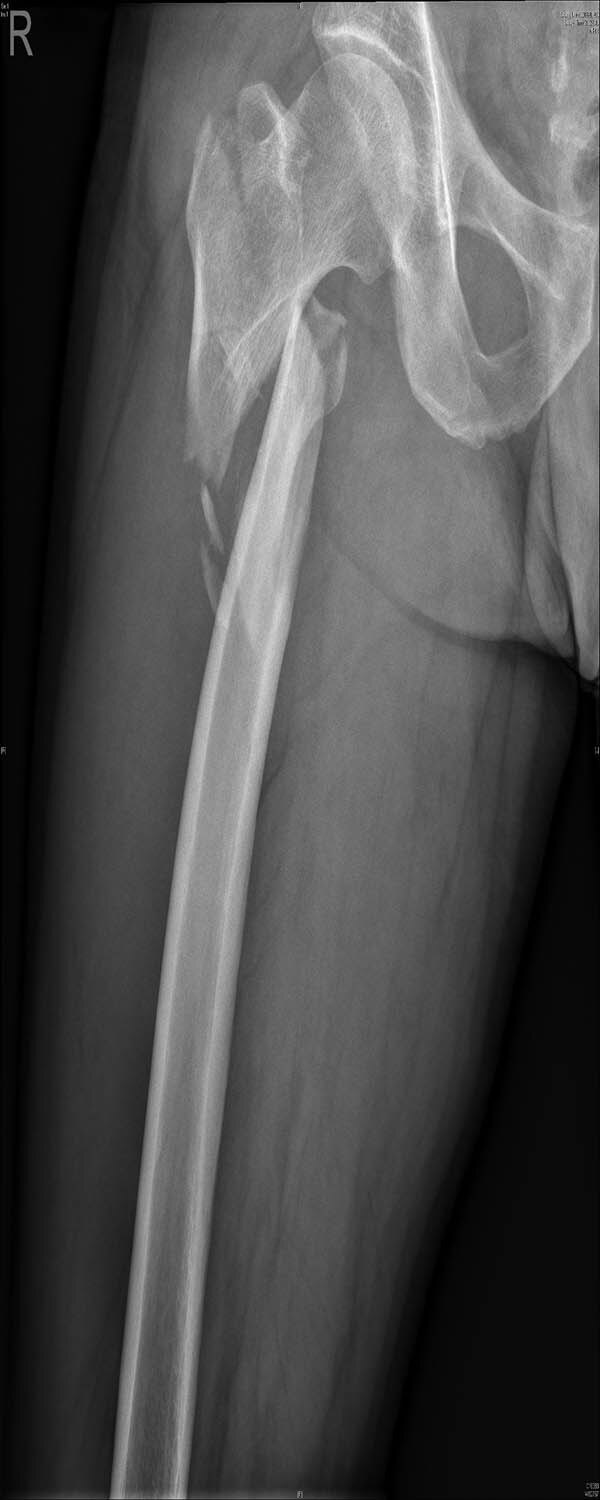
A 42-year-old man fell from height and his X-ray examination revealed that a femoral intertrochanteric fracture had occurred and that the fracture was displaced.

**Figure 3:**
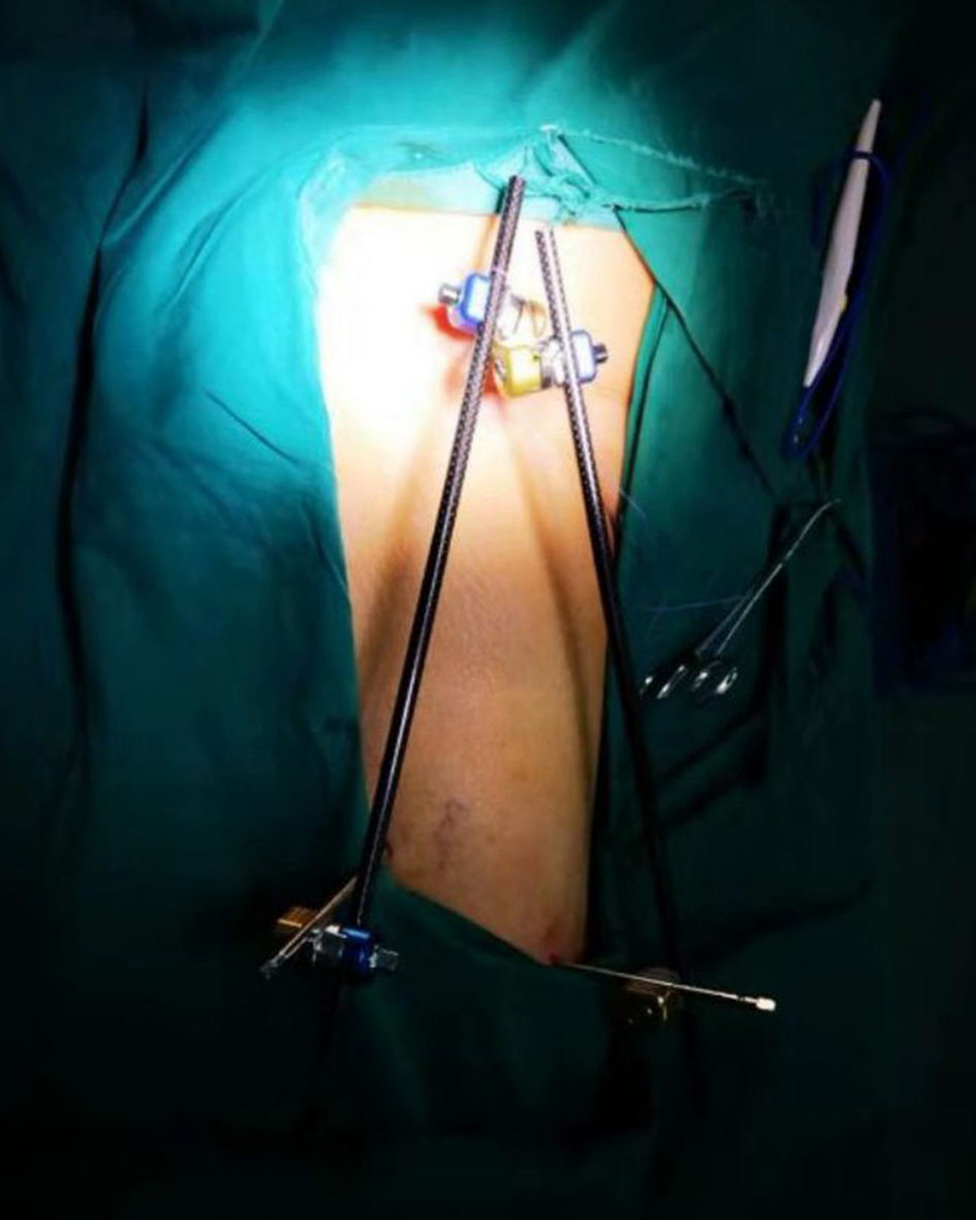
A patient with femoral intertrochanteric fracture was fitted with an external fixator during emergency surgery.

**Figure 4:**
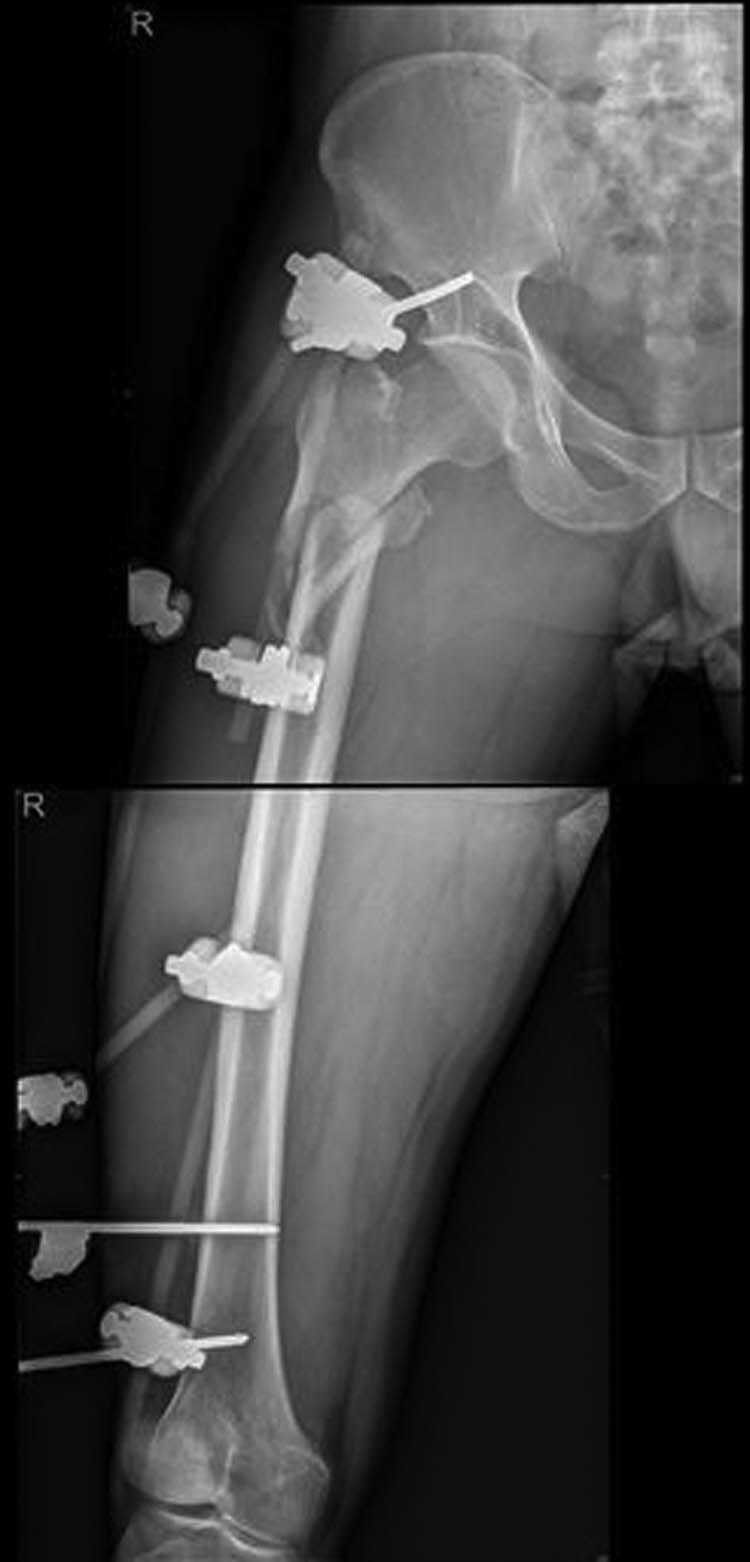
Postoperative X-ray showing good alignment following external fixation.

**Figure 5:**
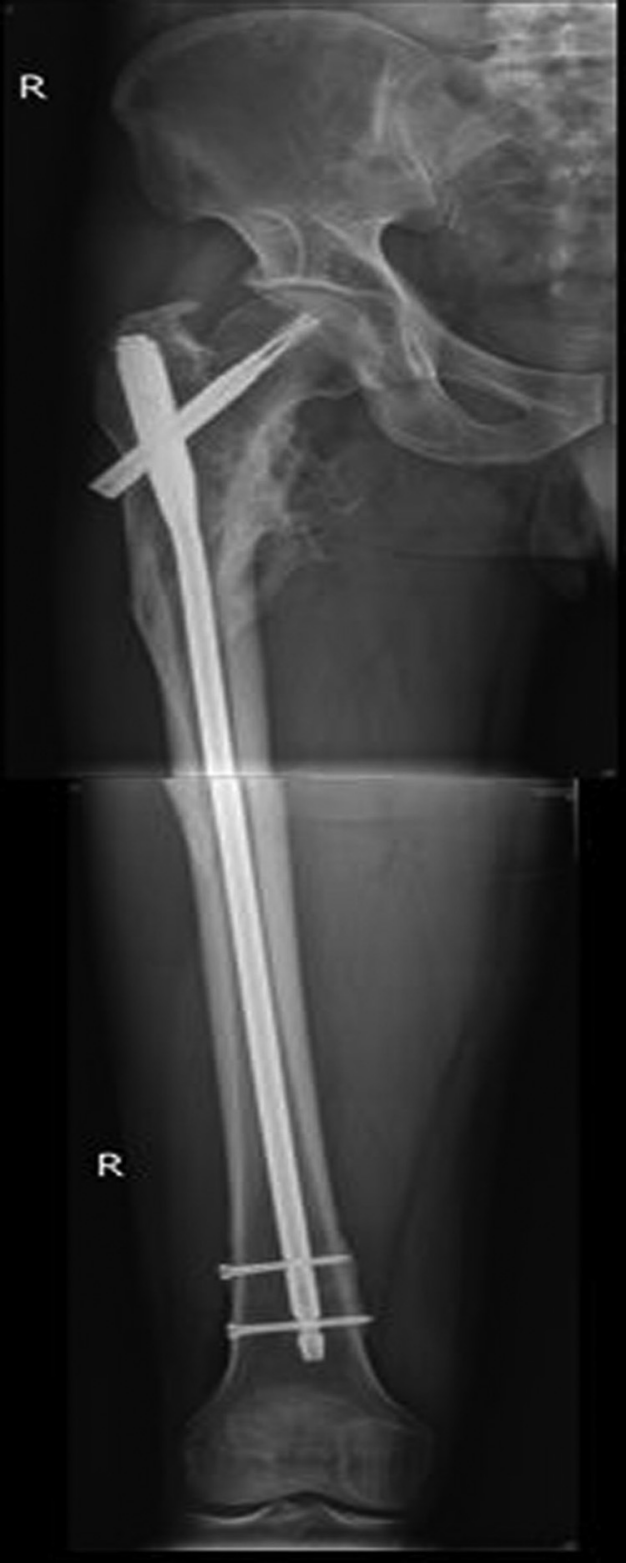
Patient with femoral intertrochanteric fracture accepted intramedullary nailing and the fracture eventually healed.

External fixation via the AIIS and distal femur was performed by a single surgeon under general anesthesia without regional nerve blockade during emergency surgery. External fixators derived from AO (Synthes, Ltd., Paoli, PA), Trauson (Trauson, Ltd., Changzhou), and Carefix (Carefix, Ltd., Shanghai) were used. Patients were placed in a supine position on the operating table. A C-arm was used to locate the points of the AIIS, and a minimal incision of 1–2 cm in length was made. The deep fascia of the pelvis and femur were incised. Proximally, the lateral femoral cutaneous nerve was protected by a surgical hook. One pin (diameter 6.0 mm, length 180 mm, thread length 60 mm) was placed at the AIIS on the same side as the PFF. Distally, two crossing pins (diameter 6.0 mm, length 180 mm, thread length 60 mm) were placed at the distal femur through two minimal incisions. High-strength and fully transparent carbon fiber rods (diameter 8 mm, length 400 mm) were fixed, manipulative reduction was performed along the strength line under the C-arm, and the clamps were tightened. The incisions were then sutured. A typical case is presented in Figures 2–5.

### 2.3 Postoperative care

Postoperatively, the pin tract of external fixation was cleaned each day. Antibiotics were used for 1–3 days if necessary in young patients who had open fractures (Gustilo I–II) to prevent postoperative infection. After external fixation, young patients were permitted to turn over regularly under careful observation to prevent bedsores. The external fixation was then removed and surgery was performed when the patients’ general and local condition tolerated conversion to intramedullary nailing, which was clinically preferred as a definitive treatment.

For data collection, surgical time was defined as the time from making the skin incision to skin closure. Blood loss was calculated throughout the surgical time. The degree of pain and treatment results were scored using a visual analog scale (VAS) [18]. VAS_1_ was defined as the degree of pain after trauma, and VAS_2_ was defined as the degree of pain after external fixation via the AIIS. The fracture reduction rate (FRR) of the femur was defined as (*A − B*)/*A* × 100%, where *A* and *B* are the displacement degree of the fracture pre- and post-surgery, respectively. The FRR was used to evaluate the outcome of fracture reduction.

The use of external fixators, intramedullary nails, peri-surgical and later complications, external fixation time, and fracture healing time was recorded. Union was defined as the absence of pain and the presence of bridging calluses in three of the four cortices seen on the anteroposterior and lateral radiographic views. Patients were followed up to the bone union. The hip function was evaluated using the Harris Hip Score (HHS) [26].

### 2.4 Statistical analysis

Single-factor analysis of variance (ANOVA) was used to compare the pain relief pre- and post-surgery. Statistical analyses were performed using SAS software (version 9.4; SAS Institute). *P* < 0.01 was considered significant.

## 3 Results

External fixation via the AIIS was successfully converted to intramedullary nailing as a definitive treatment in all 23 young patients. The use of the C-arm made the AIIS location more easily, and the mean surgical time was 20.3 ± 7.3 min (range, 15–25 min). Minimal invasive external fixation procedures resulted in low blood loss (25.3 ± 10.8 mL, range 10–50 mL). The intra- or post-surgery safety of this method of external fixation was proven by the absence of iatrogenic lateral femoral cutaneous nerve palsy, femoral nerve palsy, or femoral vessel injuries. In summary, a total of 10 AO (43.5%), 5 Trauson (21.7%), and 8 Carefix (34.8%) external fixators were applied and showed excellent biomechanical stability. No failure of external fixation was observed post-surgery. Due to pin tract cleaning each day, use of antibiotics, and short external fixation time, no pin tract infection was found.

This new method of external fixation for PFF presented satisfactory clinical outcomes. First, significant pain relief was found when comparing pre- and post-external fixation surgery VAS scores. VAS_1_ was used to evaluate the degree of pain pre-surgery. A total of two cases with a score of 3–4, two cases with a score of 5–6, 18 cases with a score of 7–8, and one case with a score of nine were observed. The mean VAS_1_ was 7.0 ± 1.4. VAS_2_ was used to evaluate the pain relief post-surgery. Two cases with a score of 3–4, 11 cases with a score of 5–6, and two cases with a score of 7–8 were observed. The mean VAS_2_ was 4.7 ± 1.3. VAS_1_ and VAS_2_ were compared using single-factor ANOVA; VAS_2_ was lower than VAS_1_ (*P* < 0.01).

Second, a great degree of fracture reduction and high FRR further confirmed the effectiveness of this external fixation method. Post-surgery images were measured and compared regularly to the pre-surgery images. The mean degree of fracture reduction was 13.5 ± 6.9, which was achieved by the external fixator’s strong traction and rigid external fixation functions. As a result, the major displacement of the PFF was reduced and the mean FRR of the proximal femur was 58.1 ± 17.0. All patients had good performance after external fixation via the AIIS for decreased pain intensity and early mobilization post-surgery, which placed patients at low risk of bedsores, pneumonia, deep vein thrombosis, and urinary tract infection. No complications were observed after external fixation and before intramedullary nailing.

Third, the condition of all patients improved and they accepted intramedullary nailing as the definitive treatment after a mean of 7.6 ± 4.0 days (range, 5–21 days). Being different from previous external fixation methods, both the greater trochanter and medullary cavity of the femur were not interfered by this technique, which facilitated the intramedullary nailing appliance. Early mobilization was encouraged 1 day after intramedullary nailing.

Finally, the mean hospital stay was 28.7 ± 8.7 days (range, 19–56 days; Table 2) and the mean follow-up was 23.5 ± 7.9 months (range, 12–36 months), whereas the mean healing time was 22.8 ± 5.7 weeks (range, 12–32 weeks). At the final follow-up, all patients had an X-ray examination and femoral axial alignment evaluation. Completely normal alignment was present, and varus angulation >5° was observed in 2 (8.6%) patients with limb shortening of 0.5 ± 0.2 cm (range, 0.45–0.80 cm). The HHS indicated that 12, 8, and 3 patients exhibited excellent, good, and fair results, respectively (Table 3).

**Table 2:** Surgical characteristics

Surgical characteristics	Mean ± SD/*n* (%)
Surgical time (min)	20.3 ± 7.3
Blood loss (mL)	25.3 ± 10.8
VAS_1_ scores	7.0 ± 1.4
VAS_2_ scores	4.7 ± 1.3^a^
Fracture reduction (%)	58.1 ± 17.0
Fracture reduction degree (°)	13.5 ± 6.9
Failure of external fixation	0
Iatrogenic nerve palsy	0
Iatrogenic vessel injury	0
Pin tract infection	0
External fixation time (days)	7.6 ± 4.0
Intramedullary nailing	23
Hospital time (days)	28.7 ± 8.7

a *P* < 0.01, VAS_2_ vs VAS_1_; values are expressed as the mean ± standard deviation or *n* (%).

**Table 3:** Follow up and functional outcome

Followup and results	Mean ± SD/*n* (%)
Follow up (months)	23.5 ± 7.9
Healing time (weeks)	22.8 ± 5.7
Shortening (cm)	0.5 ± 0.2
Varus angulation (>5°)	2 (8.6%)
Harris hip score	
Excellent/good/fair	12/8/3

Values are expressed as the mean ± standard deviation or *n* (%).

## 4 Discussion

With the development of the transportation and construction industries, high-energy trauma to the hip is exhibiting an increasing trend. This trauma mainly occurs in young patients, characterized by major fracture displacement, heavy pain, and open fractures [1,2]. Multisystem trauma in young patients is another challenge for trauma surgeons in emergency care.

Considering the aforementioned factors, acute treatment of PFFs in young patients should be different from treatment in the elderly, requiring more patience from the trauma surgeons in clinical practice. Skin or skeletal traction is traditionally used for initial stabilization of the femur [3,4,5,18] and then converted to intramedullary nailing as reliable internal fixation in a two-stage process [2,23] when the patient’s general condition tolerates major surgery in a few days. However, uncontrolled pain management, unsatisfied fracture reduction, and no permission for post-surgery mobilization cause traction methods unacceptable by young trauma patients.

A variety of external fixators have been designed for elderly patients, severely injured patients, and open fractures in young patients suffering PFFs and applied as a definitive treatment due to poor general or regional conditions. Previous studies have identified advantages of external fixators applied in elderly patients, which include low surgical risk, early mobilization, and reduced hospital stay [17,27,28,29]. A previous study [11,12] described a pelvifemoral external fixator indicated for acute treatment of explosive injuries that provides stable bone fixation and allows early mobilization. However, pin-related complications and biomechanical instability are inevitable shortcomings of external fixators in clinical practice [16,17]. Moreover, external fixation cannot be converted clinically to intramedullary nailing as a reliable surgical procedure for PFF [2,23]. With the development of EARS [21], young patients have high requirements for satisfactory fracture reduction, continuous pain control, and early post-surgery mobilization. Therefore, exploring an ideal method of external fixation prior to intramedullary nailing is important for treating PFF in young trauma patients.

Our previous anatomical study of the pelvis revealed that the bony tract from the AIIS to the posterior superior iliac spine provides an ideal location for external fixation pins with a strong anti-pulling force [22]. The osseous passage from the AIIS to the posterior superior iliac spine provides an effective holding force for the pin, counteracting the femoral displacement, and making the external fixation system more biomechanically stable. A large contact surface between the pins and the bone channel and a degree of controlled sliding contribute to the mechanical stability of the external fixation. Taking this into account, a novel effective method for treating PFF was developed in this study.

In this study, external fixation via the AIIS was successfully performed in all patients and shown to be a safe, rapid, and effective treatment protocol in an emergency for trauma surgeons. The mean surgical time was only 20 min and blood loss was only 25 mL. No complications, including iatrogenic nerve and vascular injuries, pin tract infection, or pin loosening, were noted.

Pin tract infection is a common clinical complication that requires daily cleaning with antiseptic solutions, oral antibiotics, and even removal of the pins. Vossinakis and Badras [13] reported that 16% of patients develop superficial pin tract infections. Karn et al. [16] reported pin tract infection in 60% of cases, and all healed after removal of the pins. Kazemian et al. [4] found that 70% of elderly patients with intertrochanteric fractures suffer superficial pin tract infection and 30% suffer deep infection. This was not observed in our study, possibly due to the short external fixation time and anti-infection procedures.

Pain management is of particular importance in patients with PPF, as uncontrolled pain may result in severe clinical outcomes and delayed recovery [30]. Kazemian et al. [4] found no difference between skeletal traction and external fixation in patients with intertrochanteric fractures (*P* > 0.05). As a result, external fixation via the AIIS was a useful protocol offering significant pain relief. The post-surgery pain was found to be easily controllable, making the care and mobilization of these patients easier.

The FRR was used to evaluate the degree of fracture reduction. This novel method of external fixation could initially correct ∼60% of the fracture displacement of the femur, and anatomic or almost anatomic reduction was achieved using intramedullary nailing as a definitive treatment. However, Kazemian et al. [4] reported that only 26% of patients with skeletal traction achieved acceptable reduction, and 86% of patients with external fixation had the same result.

Shortening and varus angulation are well-recognized complications of external fixation in unstable fractures or in the presence of severe osteoporosis [13,16,17]. Vossinakis and Badras [13] reported that 12 (27%) patients developed shortening of 18 mm (15–30 mm). In a previous report [16], 12 (24%) patients had an average shortening of 14 mm (range, 5–20 mm) and 10 patients had a varus angulation of 5° (range, 4–8°). Arslan et al. [17] reported a compression pin technique that demonstrated the rate of varus angulation as >5° for 20% of patients. In our study, only 2 (8.6%) patients had varus angulation (>5°) and limb shortening (<1 cm) according to the X-ray evaluation at final follow-up. This is due to early good reduction by an external fixator via the AIIS, which facilitates conversion to intramedullary nailing.

Bedsores are common complications of long-term bed rest for uncontrolled pain and inadequate stabilization [4,20]. Kazemian et al. [4] found that as many as 30% of intertrochanteric fracture patients suffer bedsores, and it was not significantly different between skeletal traction and external fixation (*P* > 0.05). Our study did not find bedsores or other complications (pneumonia, deep vein thrombosis, and urinary tract infection). This result was much better than previous reports [4,20], which we attribute to continuous pain management, short external fixation time, and early mobilization.

Previous studies revealed that a major complication of external fixators is a decrease in the range of motion of the hip and knee joints, necessitating removal of the fixators and physiotherapy [4,13,14,31]. Kazemian et al. [4] found that 30% of patients with intertrochanteric fractures suffer from the decreased motion of the hip and knee joints. This complication was not encountered in this study. This may be attributed to short external fixation time, adequate stabilization, and early joint mobilization.

All patients were followed up for >12 months until bone union was achieved. Functional outcomes wereevaluated by HHS [26]; ∼90% patients achieved excellent or good results (score >90 score), which was better than skeletal traction (score = 57) and external fixation (score = 66) [4].

Although previous studies reported the use of external fixators in osteoporosis patients and achieved good results [13,14,15,16,17,29], patients with osteoporosis were excluded from this study, as local pin holding and crossing-joint frame bridging are likely to fail in these patients. Amini et al. [1] reported that 13.5% of young patients with PFFs had major complications requiring revision surgery, but we did not find that in this study.

In addition, patients with severe soft tissue injuries (Gustilo III) were not indicated for intramedullary nailing due to a high risk of infection [11,12]. For high-energy hip trauma, Dingemans et al. [32] compared direct intramedullary nailing and two-stage treatment, concluding that two-stage treatment is a safe treatment option in young patients in terms of post-surgery wound infections and union rates. Our results are similar and support this opinion.

Despite these advantages of external fixation via the AIIS, Biz et al. [5] pointed out that skin or skeletal traction for patients with PFFs should be discouraged as standard practice, which is widely supported in the international literature. External fixation via the AIIS is an alternative surgical procedure for acute treatment of PFF in young trauma patients. However, this study has certain limitations. First, a small number of patients were evaluated. Second, patients were followed up for a relatively short period of time. Third, a control group was not set up to compare results. Long-term investigations and larger patient groups are required to validate the findings.

## 5 Conclusion

In conclusion, this study revealed that external fixation via the AIIS is a feasible, safe, and effective surgical technique when applied appropriately. The technique is valuable and should be considered for acute treatment of PFF in young trauma patients.
